# Inheritance patterns of plumage coloration in common buzzards *Buteo buteo* do not support a one-locus two-allele model

**DOI:** 10.1098/rsbl.2018.0007

**Published:** 2018-04-18

**Authors:** Elena Frederika Kappers, Christiaan de Vries, Anneke Alberda, Wolfgang Forstmeier, Christiaan Both, Bart Kempenaers

**Affiliations:** 1 Conservation Ecology Group, Groningen Institute for Evolutionary Life Sciences, University of Groningen, Groningen, The Netherlands; 2 Department of Behavioural Ecology and Evolutionary Genetics, Max Planck Institute for Ornithology, Seewiesen, Germany; 3 Noormanstrjitte 30, Wijnjewoude, The Netherlands

**Keywords:** animal model, heritability, inheritance, colour polymorphism, common buzzard, *Buteo buteo*

## Abstract

Balancing selection is a major mechanism to maintain colour polymorphisms over evolutionary time. In common buzzards, variation in plumage colour was reportedly maintained by a heterozygote advantage: heterozygote intermediates had higher fitness than homozygote light and dark morphs. Here, we challenge one of the basic premises of the heterozygote advantage hypothesis, by testing whether plumage colour variation in common buzzards follows a one-locus two-allele inheritance model. Using a long-term population study with 202 families, we show that colour variation in buzzards is highly heritable. However, we find no support for a simple Mendelian one-locus two-allele model of inheritance. Our results rather suggest that buzzard plumage colour should be considered a quantitative polygenic trait. As a consequence, it is unlikely that the proposed heterozygote advantage is the mechanism that maintains this genetic variation. We hypothesize that plumage colour effects on fitness might depend on the environment, but this remains to be tested.

## 1. Introduction

One of the big questions in biology is how genetic variation is maintained in populations over evolutionary time. Some proposed mechanisms involve balancing selection with a form of frequency-dependent feedback, resulting in fitness benefits to the rare allele [1]. Another form of balancing selection is overdominance, where heterozygotes have higher fitness than both homozygotes, but relatively few examples exist in natural populations [2,3].

A prime example of suggested overdominance in nature concerns the colour polymorphism observed in common buzzards *Buteo buteo* [4]. Colour polymorphisms are relatively common in raptors [5–8] and typically involve variation in the amount of melanization. Some evidence suggests that this trait variation is determined by simple Mendelian inheritance [9].

Common buzzard plumage varies along a light–dark continuum, but has been categorized into three morphs [10]: light, intermediate and dark. Parent–offspring resemblance was consistent with a one-locus two-allele model, whereby intermediates (supposedly the heterozygotes) had higher fitness than light and dark morphs (supposedly the homozygotes; [4]). However, this conclusion of simple Mendelian inheritance with a one-locus two-allele model was based on sparse data: overall 162 offspring with *n* < 5 offspring for half of the parental combinations [4]. In two other buteonine raptors, ferruginous hawks *B. regalis* and Swainson's hawks *B. swainsoni*, similar patterns of inheritance have been suggested [5,6], but no heterozygote advantage was found in Swainson's hawks [11]. However, also in these studies, inheritance patterns were derived from exiguous sample sizes (*n* = 5 offspring for one of the three possible parental combinations in [5]; *n* < 8 offspring for three of the four parental combinations in [6]).

Our study aims to re-examine the hypothesis that morph variation in common buzzards can be explained by a one-locus two-allele model. We tested whether the proportions of offspring of the different morphs produced by parents of known morph followed the predicted frequencies of a simple Mendelian trait. As an alternative, we examined whether the observed variation can be explained assuming polygenic inheritance with more continuous trait variation. To this end, we used our pedigree to calculate the heritability of plumage colour (i.e. the proportion of phenotypic variance explained by additive genetic variance), using a seven-morph plumage scale that better captures continuous variation [10].

## 2. Material and methods

### (a) Study population, colour score and pedigree information

Data on common buzzards come from a long-term population study in Friesland, The Netherlands, started in 1996 (see the electronic supplementary material, appendix S1). Since 2001, all breeding common buzzards and their 18–53 day old offspring (mean 33.8 days ± 3.6 s.d.) were colour-scored by one observer (CdV), using a seven-morph scale ranging from very dark to very light [10]. Juvenile plumage colour does not change substantially later in life (repeatability: *r* > 0.74; [10]).

We assembled a two-generation pedigree of 1279 birds, including 989 juveniles scored as fledglings between 2001 and 2016, and their 292 parents. The pedigree was based on field observations (i.e. direct sightings, photographs, captures and identification based on moulting feathers), assuming strict monogamy. There is no evidence for intraspecific brood parasitism in buzzards and extra-pair paternity (EPP) is presumably rare. EPP levels reported in other socially monogamous raptors are low (for a review, see table 1 in [12]) and in a related *Buteo* species, 5% of the offspring were extra-pair [13]. Previous work showed that EPP has a negligible impact on quantitative genetic estimates if the EPP level is low (less than 20% of offspring) and if sample sizes are sufficiently large [14]. Fathers produced on average 6.7 (median 5; range 1–31) and mothers 6.5 (median 4; range 1–31) offspring during the study period. In total, 976 mother–offspring relationships, 978 father–offspring relationships, 4157 full-sibling links and 10 869 half-sibling links were informative for the heritability analysis. Pedigree statistics were performed using the R package pedantics [15].

### (b) The inheritance pattern of colour morph

To examine the one-locus two-allele model of inheritance, we repeated the analysis presented in [4]. First, we converted our seven-morph scheme into the three-morph scheme (light, dark, intermediate) that best approached the previous classification [10]. As scoring schemes could not be perfectly matched, we examined four alternative scenarios of lumping individuals into the three-morph scheme (see the electronic supplementary material, appendix S2). The expected offspring morph frequencies were solely based on the phenotypes of both parents (table 1). We used a Pearson's *χ*^2^ exact test on counts in StatXact (v. 4) to compare observed frequencies between parental combinations or between studies.

**Table 1.** Inheritance of plumage colour morph in common buzzards from Friesland, The Netherlands. Morph classes are dark (*D*), intermediate (*I*) and light (*L*) scored under scenario 1 (see the electronic supplementary material, appendix S2). Observed morph shows percentage of offspring of each parental combination. Expected morph is the percentage of offspring of each morph expected under a one-locus two-allele model with intermediates being heterozygote. *N*_offspring_ indicates total number of offspring from each parental combination. Italic value highlights overrepresented categories.

		observed morph (%)			expected morph (%)		
parents	*N*_offspring_	*D*	*I*	*L*	*D*	*I*	*L*
*D* × *D*	97	83.5	*16.5*	0	100	0	0
*D* × *I*	350	47.1	48.3	*4.6*	50	50	0
*D* × *L*	32	*18*.*7*	43.8	*37.5*	0	100	0
*I* × *I*	258	15.1	*74*	10.9	25	50	25
*I* × *L*	138	*2.9*	31.9	*65.2*	0	50	50
*L* × *L*	94	*1.1*	*14.9*	84	0	0	100

### (c) Heritability of plumage colour

We estimated the heritability of plumage colour (using the seven morphs) with quantitative genetic methods, assuming continuous variation. We constructed a linear mixed effect model incorporating relatedness information (‘animal model’ [16]) to partition phenotypic variance into autosomal additive genetic variance and environmental variance. As random effects, we included birth year (to account for annual fluctuations in environmental conditions), nest (to account for shared natal environment), and mother and father identity. In all analyses, we combined data from female and male offspring and we initially included offspring sex as a fixed effect. Because this effect was not significant, we excluded it in the final models. We fitted the animal model using a Bayesian framework implemented in R (version 3.3, [17]) with the package MCMCglmm [18]. We chose weakly informative priors (inverse-Gamma distribution with *ν* = 0.002 and *V* = 1). Models were sampled every 10 iterations, with an initial burn-in of 100 000, for 1 000 000 samples, which resulted in autocorrelation less than 0.05 for all parameters. Posterior means and 95% credible intervals were estimated across the thinned samples for the mean effect and variance ratios.

## 3. Results and discussion

In contrast to conclusions from a previous study on common buzzard morphs [4], we found no support for the one-locus two-allele model of inheritance (table 1). Across all scoring scenarios, the observed segregation deviated substantially from the expected one (table 1; electronic supplementary material, figure S1 and table S1). Most importantly, intermediate offspring were greatly overrepresented in intermediate × intermediate pairs and underrepresented in dark × light pairs. Intermediate × intermediate pairs should produce fewer intermediates (expected: 50%) than dark × light pairs (expected: 100%), but observed frequencies are significantly in the opposite direction (*p* < 0.001).

To assess why our conclusions deviate from those presented earlier [4], we compared sample sizes and observed offspring morph frequencies between the two studies (electronic supplementary material, table S2). The observed frequencies are remarkably similar and do not differ significantly even when using anti-conservative tests on count data that ignore the non-independence of offspring from the same nest or pair (all *p* ≥ 0.14 in electronic supplementary material, table S2).

Using our seven-morph classification, the animal model (model 1 in table 2) gives a heritability estimate for plumage colour of *h^2^* = 0.82 (95% CrI: 0.75–0.88). Shared nest environment and birth-year effects did not explain additional variation and neither did they alter the estimates of heritability, nor the maternal or paternal effects (model 2 in table 2). The effect of mother identity was not larger than the effect of father identity (table 2; 95% CrI of (*V*_M_–*V*_F_)/*V*_P_: −0.1–0.1), suggesting no or minimal additional maternal effects (e.g. via egg composition) on offspring plumage colour. These results, combined with the observation that colour variation in our population is rather continuous and unimodal [10], suggest that plumage colour in buzzards should be considered a quantitative polygenic trait. This is contrary to conclusions based on inheritance patterns of melanic coloration in most other bird species [9], where the melanic forms can either be dominant [5,7,19] or recessive [8,20] (but note that this includes species with two distinct morphs [8,19,20] as well as species with a more continuous colour variation [5,7]).

**Table 2.** Proportion of variances and their corresponding 95% CrI from animal models used to partition phenotypic variance (*V*_P_ = 2.24) into autosomal additive genetic (*V*_A_) and environmental components of variance (*V*_M_ = mother identity, *V*_F_ = father identity, *V*_N_ = nest, *V*_Y_ = birth year; *V*_R_ = residuals).

model	*V*_A_/*V*_P_ = *h*^2^	*V*_M_/*V*_P_	*V*_F_/*V*_P_	*V*_N_/*V*_P_	*V*_Y_/*V*_P_	*V*_R_/*V*_P_
1	0.82 (0.75–0.88)	0.06 (10^−3^–0.11)	0.05 (10^−3^–0.10)			0.08 (0.03–0.13)
2	0.81 (0.75–0.87)	0.06 (10^−3^–0.11)	0.05 (10^−3^–0.10)	0.006 (6 × 10^−5^–0.02)	0.003 (10^−3^–0.01)	0.07 (0.03–0.12)

In our buzzard population, plumage colour was highly heritable, independent of sex and not influenced by environmental factors (table 2). Quantitative genetic studies of plumage coloration in birds such as tawny owls *Strix aluco* [7], barn owls *Tyto alba* [21] and common kestrels *Falco tinnunculus* [22] showed similar high heritability values (*h*^2^ = 0.80, 0.81 and 0.67–0.83 respectively). This implies that selection can act on the trait and that the variance is either selectively neutral or a mechanism exists that keeps the polymorphism stable.

The maintenance of the colour polymorphism in common buzzards has previously been explained by heterozygote advantage (higher fitness of the intermediate morph), but the present results question this explanation. Under a one-locus two-allele model, heterozygote advantage is sufficient to maintain a stable polymorphism where both alleles should be equally common in the population. However, in a polygenic inheritance system as supported by our data, overdominance would not be an effective mechanism for maintaining many alleles at individual loci [23], and it is more likely that variation is maintained through genotype–environment interactions [24]. We suggest the testable hypothesis that the fitness effects of plumage colour are environment-dependent, which may explain geographical variation in morph frequencies [24].

## Supplementary Material

Appendices 1 and 2; Figure S1 and Tables S1 and S2

## Data accessibility

Data are available from the Open Science Framework (https://osf.io/3947z) [25].
