# Supplementary material for: Inheritance patterns of plumage coloration in common buzzards Buteo buteo do not support a one-locus two-allele model
Source: Biol Lett. 2018 Apr 18;14(4):20180007. doi: 10.1098/rsbl.2018.0007 (PMC5938563; doi:10.1098/rsbl.2018.0007)
Supplement: Appendices 1 and 2; Figure S1 and Tables S1 and S2 [file rsbl20180007supp1.doc]

# **Inheritance patterns of plumage coloration in Common Buzzards *Buteo buteo* do not support a one-locus two-allele model**

Elena Frederika Kappers^1,2*^, Christiaan de Vries^3^, Anneke Alberda^3^, Wolfgang Forstmeier^2^, Christiaan Both^1^, Bart Kempenaers^2^

^1^Conservation Ecology Group, Groningen Institute for Evolutionary Life Sciences, University of Groningen, Groningen, the Netherlands.
^2^Department of Behavioural Ecology and Evolutionary Genetics, Max Planck Institute for Ornithology, Seewiesen, Germany.
^3^Noormanstrjitte 30, Wijnjewoude, The Netherlands.
*corresponding author ([e.f.kappers@rug.nl](mailto:e.f.kappers@rug.nl))

**Appendix 1.** Study site

The study site encompasses a 5724 ha area with 1400 ha of forested patches, centred at 53°04'09.2"N, 6°13'46.6"E, and contains on average 76 ± 12 SD breeding pairs/year over a 20 year period.

**Appendix 2.** Colour morph scoring

To convert our seven-morph colour scoring scheme into the three basic morph types light, intermediate and dark, we used four different scenarios, based on [1]: (1) 1-2=dark, 3-4-5=intermediate, 6-7=light; (2) 1=dark, 2-3-4-5=intermediate, 6-7=light; (3) 1=dark, 2-3-4-5-6=intermediate, 7=light; (4) 1-2=dark, 3-4=intermediate, 5-6-7=light. The first scenario is represented in table 1 in the main text, and is based on the best fit when the authors of the original paper scored buzzard pictures that we also scored on our seven morph scale (see [1]).

**Figure S1.**

Observed and expected inheritance of plumage colour morph for all parental combinations in Common Buzzards from Friesland, The Netherlands. (a)-(d) show the results for scenarios 1-4 (see above), respectively. Bars represent percentages of offspring of each morph class (brown = dark, orange = intermediate, beige = light) observed in our study (left panel) and expected from a one-locus two-allele inheritance pattern with intermediates as heterozygotes (right panel) for every parental combination shown on the y-axis. See table 1 and S1 for sample sizes and statistical analysis.


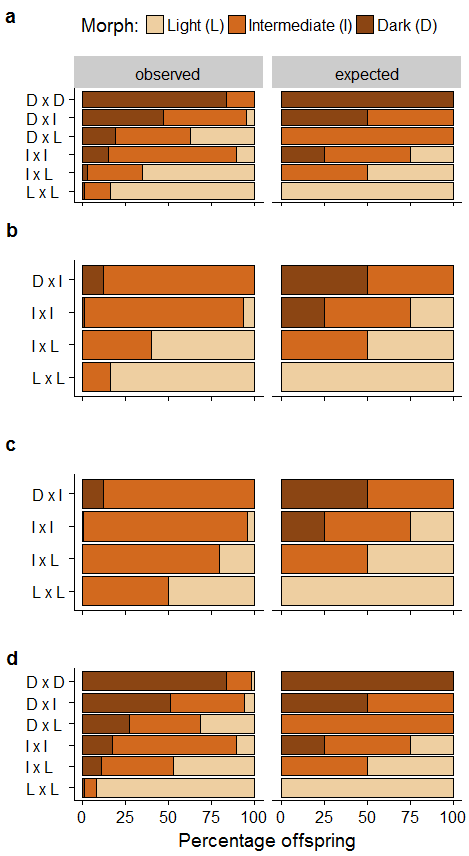


**Table S1.**

Inheritance of plumage colour morph in Common Buzzards from Friesland, The Netherlands. Morph classes are dark (D), intermediate (I) and light (L), scored under scenarios 2-4 (see above). Observed morph shows the percentage of offspring of each parental combination. Expected morph is the percentage of offspring of each morph expected under a one-locus two-allele inheritance pattern with intermediates being heterozygote. N_offspring_ indicates the total number of offspring from each parental combination. Bold print highlights overrepresented categories.

|  |  | Observed morph (%) | | |  | Expected morph (%) | | |
| --- | --- | --- | --- | --- | --- | --- | --- | --- |
| Parents | N_offspring_ | D | I | L |  | D | I | L |
| **Scenario 2** |  |  |  |  |  |  |  |  |
| D x I | 34 | 11.8 | **88.2** | 0 |  | 50 | 50 | 0 |
| I x I | 671 | 0.9 | **92.5** | 6.6 |  | 25 | 50 | 25 |
| I x L | 170 | 0 | 40 | **60** |  | 0 | 50 | 50 |
| L x L | 94 | 0 | **16** | 84 |  | 0 | 0 | 100 |
| **Scenario 3** |  |  |  |  |  |  |  |  |
| D x I | 34 | 11.8 | **88.2** | 0 |  | 50 | 50 | 0 |
| I x I | 865 | 0.7 | **95.4** | 3.9 |  | 25 | 50 | 25 |
| I x L | 64 | 0 | **79.7** | 20.3 |  | 0 | 50 | 50 |
| L x L | 6 | 0 | **50** | 50 |  | 0 | 0 | 100 |
| **Scenario 4** |  |  |  |  |  |  |  |  |
| D x D | 97 | 83.5 | **14.4** | 2.1 |  | 100 | 0 | 0 |
| D x I | 283 | 50.9 | 43.1 | **6** |  | 50 | 50 | 0 |
| D x L | 99 | **27.3** | 41.4 | **31.3** |  | 0 | 100 | 0 |
| I x I | 159 | 17 | **72.3** | 10.7 |  | 25 | 50 | 25 |
| I x L | 136 | **11** | 41.9 | 47.1 |  | 0 | 50 | 50 |
| L x L | 195 | **1** | **7.2** | 91.8 |  | 0 | 0 | 100 |

**Table S2.**

Observed inheritance of plumage colour morph in Common Buzzards from Friesland, The Netherlands (our study), and from a previous study in Eastern Westphalia, Germany [2]. Morph classes are dark (D), intermediate (I) and light (L), scored based on scenario 1 (see above). Observed morph shows the percentage of offspring of each parental combination. N_offspring_ indicates the total number of offspring from each parental combination. *P-*values are based on Pearson’s chi-square exact test performed on counts for 2 x 3 tables in StatXact 4.0.

|  | Our study | | | |  | Previous study [2] | | | |  |
| --- | --- | --- | --- | --- | --- | --- | --- | --- | --- | --- |
| Parents | N_offspring_ | Observed morph (%) | | |  | N_offspring_ | Observed morph (%) | | |  |
|  |  | D | I | L |  |  | D | I | L | *p*-value |
| D x D | 97 | 83.5 | 16.5 | 0 |  | 2 | 100 | 0 | 0 | 1 |
| D x I | 350 | 47.1 | 48.3 | 4.6 |  | 22 | 36.4 | 64.6 | 0 | 0.2531 |
| D x L | 32 | 18.8 | 43.8 | 37.5 |  | 4 | 0 | 100 | 0 | 0.1490 |
| I x I | 258 | 15.1 | 74 | 10.9 |  | 90 | 22.2 | 64.4 | 13.3 | 0.2134 |
| I x L | 138 | 2.9 | 31.9 | 65.2 |  | 41 | 2.4 | 48.8 | 48.8 | 0.1447 |
| L x L | 94 | 1.1 | 14.9 | 84 |  | 3 | 0 | 0 | 100 | 1 |

**Appendix 3.** References

1. Kappers EF, Chakarov N, Krüger O, Mueller AK, Valcu M, Kempenaers B, Both C. 2017 Classification and Temporal Stability of Plumage Variation in Common Buzzards. *Ardea* **105**, 125–136. (doi:10.5253/arde.v105i2.a1)

2. Krüger O, Lindström J, Amos W. 2001 Maladaptive mate choice maintained by heterozygoe advantage. *Evolution (N. Y).* **55**, 1207–1214.
